# Supplementary material for: Alteration of Mitochondrial Transcript Expression in Arabidopsis thaliana Using a Custom-Made Library of Pentatricopeptide Repeat Proteins
Source: Int J Mol Sci. 2023 Aug 26;24(17):13233. doi: 10.3390/ijms241713233 (PMC10487680; doi:10.3390/ijms241713233)
Supplement: Supplementary file 1 [file ijms-24-13233-s001.zip › ijms-2548190-supplementary.pdf]

Supplementary Materials

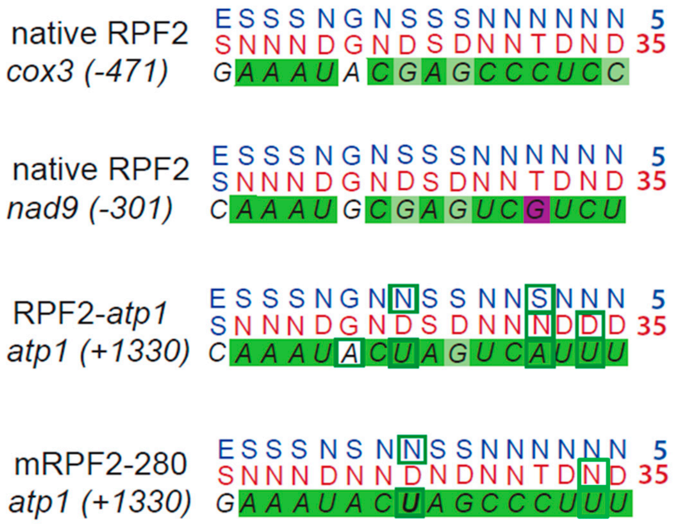

Figure S1. Binding predictions for the native RPF2, RPF2-*atp1* and transformant #280 (mRPF2-280) proteins on their respective targets, *cox3*, *nad9* or *atp1*. The amino acids modified in RPF2-*atp1* and transformant #280 are in green squares.

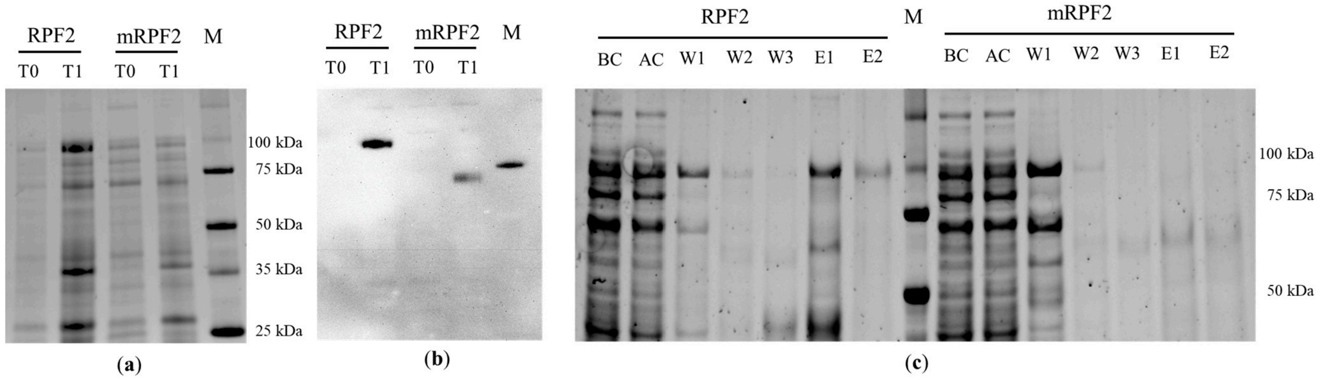

Figure S2. RPF2 and mRPF2 protein expression and purification. (**a**) SDS-PAGE showing the expression of RPF2 and mRPF2; (**b**) A western blot of the gel was probed with an anti His-tag antibody. The RPF2 construct is cloned in pETM40, adding a maltose-binding protein (MBP) tag (30 kDa) compared with the mRPF2 construct (cloned in pETM20). Thus, the expected sizes for RPF2 and mRPF2 are approximately 100 kDa and 70 kDa, respectively. T0, T1 represent the samples before and after IPTG induction, and M the size marker; (**c**) Protein purification. The samples were collected before (BC) and after (AC) passing through the Ni-NTA column, after three washes (W1, W2 and W3) and two elution steps in 50 mM HEPES-KOH, 500 mM NaCl, 10% glycerol containing 250 (E1) and 375 mM (E2) imidazole respectively.

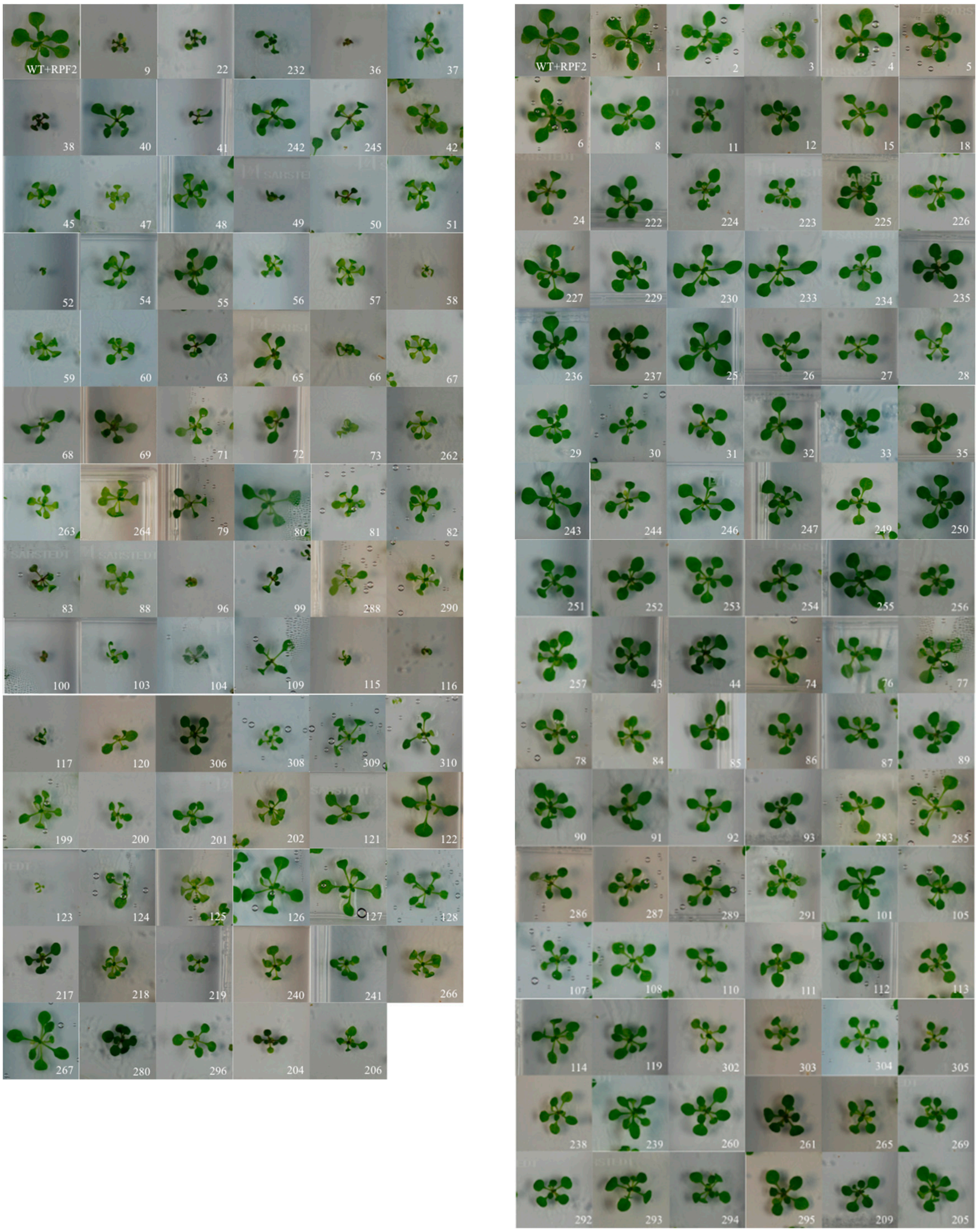

(**a**) (**b**)

Figure S3. Phenotype of plants transformed with the RPF2 library after 2 weeks on agar plates. (**a**) Eighty-two transformants with altered phenotypes compared to WT; (**b**) Ninety-five transformants with phenotype similar to WT.

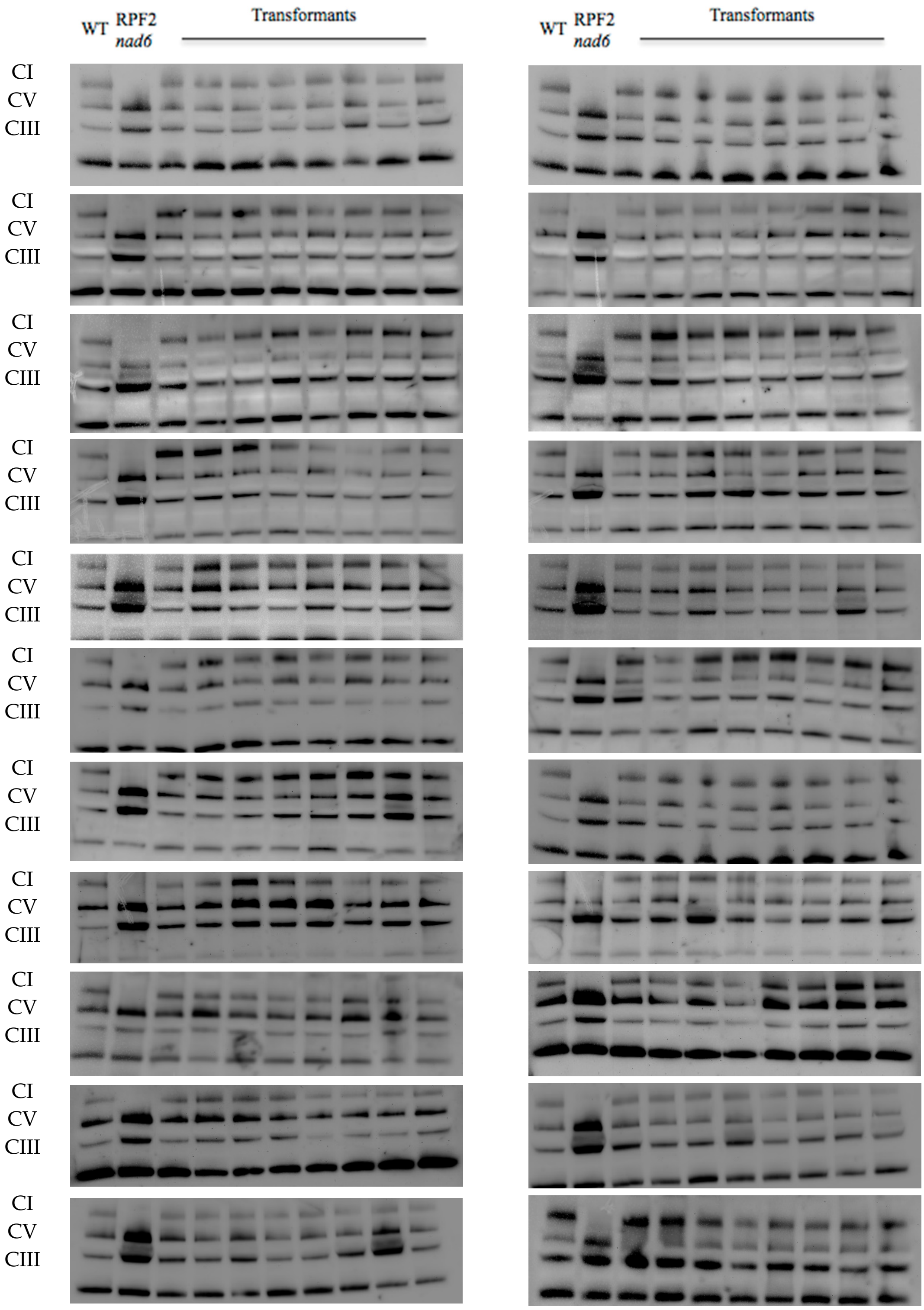

Figure S4. Analysis of the mitochondrial respiratory complexes. Western blots of mitochondrial complexes (CI, CIII and CV) separated by BN-PAGE from 177 transformants compared to WT and RPF2-*nad6*. Transformant 280 is marked with an asterisk (*).

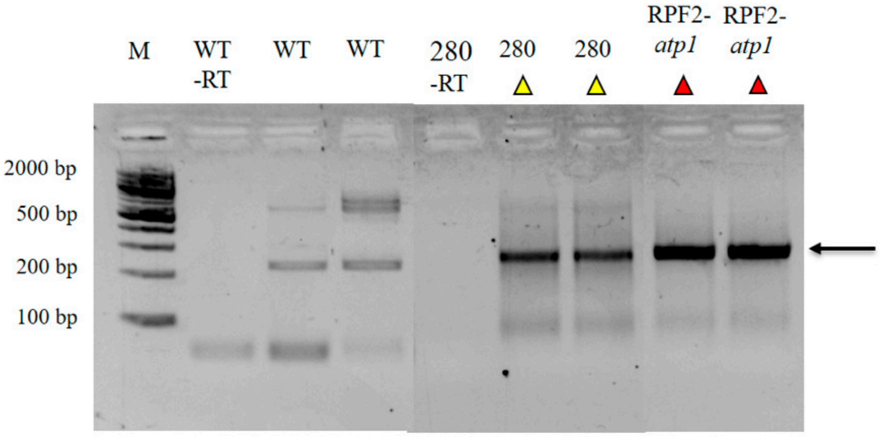

Figure S5. cRT-PCR of leaves from WT, RPF2-*atp1* and transformant #280 plants. cRT-PCR products from the uncleaved fragment and 3′ fragment on a 2% low melting agarose gel. -RT control is a cDNA synthesis reaction where the reverse transcriptase was omitted, showing the effectiveness of the DNase treatment performed before the cDNA synthesis. The arrow indicates the cRT-PCR products that were purified and sequenced. The red and yellow triangles indicate the 5′ends of the cleavage products of RPF2-*atp1* and transformant #280, respectively.

Table S1. gBlocks Gene Fragments using the 16 mRPF2 PPR motifs.

Fragment	Sequence
**1**	AATCTTTATTTTCAGGGCGCCATGGGTAATGCTG-<br>CAACTGTTTCTCCCTCTTTTTCCTTTTTCTGGAGACGAGCTTTCTCTGGTAAGACTAGT<br>TATGATTACAGAGAGAAACTGAGTAGAAATGGGCTGAGTGAATTAAAGTTA-<br>GATGAC-<br>GCTGTTGCTCTGTTCGGTGAAATGGTCAAGTCTCGTCCCTTCCCTTCCATCATTGAGT<br>TCAGCAAACTGTTGAGTGCAATTGCTAAGATGAACAAGTTCGATGTT-<br>GTCATCTCTCTCGGCGAGCAGATGCAAAACTTGGGAATTCCTCAT**RAT**CACTATACT<br>TAC**ART**ATTTTGATTAACTGTTTTTGTCGACGCTCTCAACTCCCTCTT-<br>GCTTTAGCTGTTCTTGGCAAGATGATGAAACTTGGCTATGAGCCCAATATTGTCACG<br>CTTTCTTCGCTGCTCAATGGG
**2**	ATTGTCACGCTTTCTTCGCTGCTCAATGGGTATTGCCACAGTAAGAGGA-<br>TATCCGAGGCTG-<br>TAGCTTTGGTTGATCAGATGTTCGTAACGGGGTATCAACCCAATACCGTCACATTTA<br>ACACTCTAATCCATGGACTTTTTCTTCACAACAAAGCTTCAGAA-<br>GCAATGGCTTTAATT-<br>GATCGAATGGTTGCAAAAGGTTGTCAACCA**RAT**CTGGTTACTTAT**ART**GTGGTAGTA<br>AATGGATTATGTAAGAGAGGTGATACTGATTTGGCTTTTAATCTGCTCAACAA-<br>GATGGAACAAGGGAAATTAGAACCT**AAT**GTTTTGATCTACAACACAATCATTGATG<br>GT
**3**	GTTTTGATCTACAACACAATCATTGATGGTCTTTGCAAATACAAACAT-<br>ATGGATGATGCGCTCAACCTATTTAAAGAAATGGAAACAAAAGGCATTAGACCAA<br>ATGTTGTTACCTACAGCTCCCTCATAAGTTGCCTTTGTAATTACGGAAGATGGAG-<br>TGATGCCTCTCGCCTCCTTAGTGATATGATTGAGAGGAAAATCAACCCT**RAT**GTATT<br>CACTTTC**ARC**GCATTGATCGATGCGTTTGTGAAGGAGGGCAAGCTT-<br>GTAGAGGCTGA-<br>GAAATTGTACGACGAGATGGTCAAAAGGTCCATAGATCCT**AAT**ATTGTCACATACA<br>GTTCATTGATCAACGGG
**4**	ATTGTCACATACAGTTCATTGATCAACGGGTTTTGTATGCACGATCGTCTAGAC-<br>GAGGCCAA-<br>GCAGATGTTTGAGTTCATGGTTAGCAAGCATTGTTTTCCAGATGTAGTGACTTATAAT<br>ACGCTTATAAAGGGATTTTGCAAGTATAAAAGGGTAGAAGAGGG-<br>TATGGAAGTCTTCCGTGA-<br>GATGTCTCAAAGAGGATTGGTTGGC**RAC**ACTGTCACTTAC**ARC**ATTCTTATCCAAGG<br>GTTATTTCAAGCTGGAGATTGTGATATGGCTCAAGAAATATTCAAAGAGATGG-<br>TATCTGATGGCGTGCCTCCCAATATTATGACATACAACACTTTGTTAGATGGA
**5**	ATTATGACATACAACACTTTGTTAGATGGACTTTGTAAAAATGGGAAGCTAGA-<br>GAAA-<br>GCAATGGTTGTATTCGAGTATCTGCAAAGGAGTAAAATGGAACCTACTATTTACACA<br>TATAATATTATGATTGAAGGGATGTGCAAGGCAGGGAAGGTGGAAGATGGTGG-<br>GATCTATTTTGTAATCTCAGCCTTAAAGGAGTGAAGCCG**RAT**GTTGTAGCGTAC**ART**<br>ACAATGATATCAGGATTTTGTAGGAAAGGTTCAAAGGAGGAA-<br>GCAGATGCCTTATTTAAA-<br>GAAATGAAAGAAGATGGGACTCTTCCAAACAGCGGTTGCTATAATACGCTGATTAG<br>GGCA

Table S2. P motif combinations at positions 5 and 35 in 90 mRPF2 variants along with predicted target. For each variant, positions 5 and 35 are shown in rows 1 and 2 and aligned to the predicted target (bold characters, row 3). The residues identical to mRPF2 are in light yellow boxes; amino acids and targets that differ from mRPF2 are in brown boxes.

Motifs	P1	P2	P3	P4	P5	P6	P7	P8	P9	P10	P11	P12	P13	P14	P15	P16	Predicted target
	E	S	S	S	N	S	N	S	S	S	N	N	N	N	N	N	
**mRPF2**	S	N	N	N	D	N	N	D	N	D	N	N	T	D	N	D	*nad6*
	**C**	**A**	**A**	**A**	**U**	**A**	**C**	**G**	**A**	**G**	**C**	**C**	**C**	**T**	**C**	**U**	
	E	S	N	S	N	S	N	N	N	S	N	S	N	N	N	N	
**1**	S	D	N	N	D	N	N	D	N	D	D	N	T	D	D	D	*nad7*
	**C**	**G**	**C**	**A**	**U**	**A**	**C**	**U**	**C**	**G**	**U**	**A**	**C**	**U**	**U**	**U**	
	E	S	N	S	N	S	N	N	N	S	N	N	N	N	N	N	
**2**	S	D	N	N	D	N	N	D	N	D	D	N	T	N	D	D	
	**C**	**G**	**C**	**A**	**U**	**A**	**C**	**U**	**C**	**G**	**U**	**C**	**C**	**C**	**U**	**U**	
	E	S	N	S	N	S	N	N	S	S	N	S	N	N	N	N	
**3**	S	D	N	N	D	N	N	D	N	D	D	N	T	N	N	D	*rrn26*
	**C**	**G**	**C**	**A**	**U**	**A**	**C**	**U**	**A**	**G**	**U**	**A**	**C**	**C**	**C**	**U**	
	E	S	N	S	N	S	N	N	S	S	N	S	N	N	N	N	
**4**	S	D	N	N	D	N	N	D	N	D	D	N	T	D	D	D	*nad5*
	**C**	**G**	**C**	**A**	**U**	**A**	**C**	**U**	**A**	**G**	**U**	**A**	**C**	**U**	**U**	**U**	
	E	S	N	S	N	S	N	N	S	S	N	N	N	N	N	N	
**5**	S	D	N	N	D	N	N	D	N	D	D	N	T	D	D	D	
	**C**	**G**	**C**	**A**	**U**	**A**	**C**	**U**	**A**	**G**	**U**	**C**	**C**	**U**	**U**	**U**	
	E	S	N	S	N	S	N	N	S	S	N	N	N	N	N	N	
**6**	S	D	N	N	D	N	N	D	N	D	N	N	T	D	N	D	
	**C**	**G**	**C**	**A**	**U**	**A**	**C**	**U**	**A**	**G**	**C**	**C**	**C**	**U**	**C**	**U**	
	E	S	N	S	N	S	N	S	N	S	N	S	N	N	N	N	
**7**	S	D	N	N	D	N	N	D	N	D	D	N	T	D	D	D	
	**C**	**G**	**C**	**A**	**U**	**A**	**C**	**G**	**C**	**G**	**U**	**A**	**C**	**U**	**U**	**U**	
	E	S	N	S	N	S	N	S	N	S	N	S	N	N	N	N	
**8**	S	D	N	N	D	N	N	D	N	D	D	N	T	D	N	D	
	**C**	**G**	**C**	**A**	**U**	**A**	**C**	**G**	**C**	**G**	**U**	**A**	**C**	**U**	**C**	**U**	
	E	S	N	S	N	S	N	S	N	S	N	S	N	N	N	N	
**9**	S	D	N	N	D	N	N	D	N	D	N	N	T	D	D	D	
	**C**	**G**	**C**	**A**	**U**	**A**	**C**	**G**	**C**	**G**	**C**	**A**	**C**	**U**	**U**	**U**	
	E	S	N	S	N	S	N	S	S	S	N	S	N	N	N	N	
**10**	S	D	N	N	D	N	N	D	N	D	D	N	T	D	D	D	
	**C**	**G**	**C**	**A**	**U**	**A**	**C**	**G**	**A**	**G**	**U**	**A**	**C**	**U**	**U**	**U**	
	E	S	N	S	N	S	N	S	S	S	N	N	N	N	N	N	
**11**	S	D	N	N	D	N	N	D	N	D	D	N	T	N	D	D	
	**C**	**G**	**C**	**A**	**U**	**A**	**C**	**G**	**A**	**G**	**U**	**C**	**C**	**C**	**U**	**U**	
	E	S	N	S	N	S	N	S	S	S	N	N	N	N	N	N	
**12**	S	D	N	N	D	N	N	D	N	D	N	N	T	D	D	D	
	**C**	**G**	**C**	**A**	**U**	**A**	**C**	**G**	**A**	**G**	**C**	**C**	**C**	**U**	**U**	**U**	
	E	S	S	S	N	S	N	N	N	S	N	S	N	N	N	N	
**13**	S	D	N	N	D	N	N	D	N	D	D	N	T	D	D	D	
	**C**	**G**	**A**	**A**	**U**	**A**	**C**	**U**	**C**	**G**	**U**	**A**	**C**	**U**	**U**	**U**	
	E	S	S	S	N	S	N	N	N	S	N	S	N	N	N	N	
**14**	S	D	N	N	D	N	N	D	N	D	D	N	T	D	N	D	*nad7*
	**C**	**G**	**A**	**A**	**U**	**A**	**C**	**U**	**C**	**G**	**U**	**A**	**C**	**U**	**C**	**U**	
	E	S	S	S	N	S	N	N	N	S	N	S	N	N	N	N	
**15**	S	D	N	N	D	N	N	D	N	D	N	N	T	D	D	D	
	**C**	**G**	**A**	**A**	**U**	**A**	**C**	**U**	**C**	**G**	**C**	**A**	**C**	**U**	**U**	**U**	
	E	S	S	S	N	S	N	N	S	S	N	S	N	N	N	N	
**16**	S	D	N	N	D	N	N	D	N	D	D	N	T	N	D	D	*nad7*
	**C**	**G**	**A**	**A**	**U**	**A**	**C**	**U**	**A**	**G**	**U**	**A**	**C**	**C**	**U**	**U**	
	E	S	S	S	N	S	N	N	S	S	N	S	N	N	N	N	
**17**	S	D	N	N	D	N	N	D	N	D	D	N	T	D	D	D	*nad7*
	**C**	**G**	**A**	**A**	**U**	**A**	**C**	**U**	**A**	**G**	**U**	**A**	**C**	**U**	**U**	**U**	
	E	S	S	S	N	S	N	N	S	S	N	S	N	N	N	N	
**18**	S	D	N	N	D	N	N	D	N	D	D	N	T	D	N	D	
	**C**	**G**	**A**	**A**	**U**	**A**	**C**	**U**	**A**	**G**	**U**	**A**	**C**	**U**	**C**	**U**	
	E	S	S	S	N	S	N	N	S	S	N	N	N	N	N	N	
**19**	S	D	N	N	D	N	N	D	N	D	D	N	T	D	D	D	*nad7*
	**C**	**G**	**A**	**A**	**U**	**A**	**C**	**U**	**A**	**G**	**U**	**C**	**C**	**U**	**U**	**U**	
	E	S	S	S	N	S	N	N	S	S	N	S	N	N	N	N	
**20**	S	D	N	N	D	N	N	D	N	D	N	N	T	N	D	D	*nad7*
	**C**	**G**	**A**	**A**	**U**	**A**	**C**	**U**	**A**	**G**	**C**	**A**	**C**	**C**	**U**	**U**	
	E	S	S	S	N	S	N	N	S	S	N	S	N	N	N	N	
**21**	S	D	N	N	D	N	N	D	N	D	N	N	T	D	D	D	*nad7*
	**C**	**G**	**A**	**A**	**U**	**A**	**C**	**U**	**A**	**G**	**C**	**A**	**C**	**U**	**U**	**U**	
	E	S	S	S	N	S	N	N	S	S	N	S	N	N	N	N	
**22**	S	D	N	N	D	N	N	D	N	D	N	N	T	D	N	D	*nad7*
	**C**	**G**	**A**	**A**	**U**	**A**	**C**	**U**	**A**	**G**	**C**	**A**	**C**	**U**	**C**	**U**	
	E	S	S	S	N	S	N	N	S	S	N	N	N	N	N	N	
**23**	S	D	N	N	D	N	N	D	N	D	N	N	T	D	D	D	
	**C**	**G**	**A**	**A**	**U**	**A**	**C**	**U**	**A**	**G**	**C**	**C**	**C**	**U**	**U**	**U**	
	E	S	S	S	N	S	N	S	N	S	N	S	N	N	N	N	
**24**	S	D	N	N	D	N	N	D	N	D	D	N	T	N	D	D	
	**C**	**G**	**A**	**A**	**U**	**A**	**C**	**G**	**C**	**G**	**U**	**A**	**C**	**C**	**U**	**U**	
	E	S	S	S	N	S	N	S	N	S	N	S	N	N	N	N	
**25**	S	D	N	N	D	N	N	D	N	D	D	N	T	N	N	D	
	**C**	**G**	**A**	**A**	**U**	**A**	**C**	**G**	**C**	**G**	**U**	**A**	**C**	**C**	**C**	**U**	
	E	S	S	S	N	S	N	S	N	S	N	S	N	N	N	N	
**26**	S	D	N	N	D	N	N	D	N	D	D	N	T	D	D	D	
	**C**	**G**	**A**	**A**	**U**	**A**	**C**	**G**	**C**	**G**	**U**	**A**	**C**	**U**	**U**	**U**	
	E	S	S	S	N	S	N	S	N	S	N	S	N	N	N	N	
**27**	S	D	N	N	D	N	N	D	N	D	D	N	T	D	N	D	
	**C**	**G**	**A**	**A**	**U**	**A**	**C**	**G**	**C**	**G**	**U**	**A**	**C**	**U**	**C**	**U**	
	E	S	S	S	N	S	N	S	N	S	N	N	N	N	N	N	
**28**	S	D	N	N	D	N	N	D	N	D	D	N	T	N	D	D	
	**C**	**G**	**A**	**A**	**U**	**A**	**C**	**G**	**C**	**G**	**U**	**C**	**C**	**C**	**U**	**U**	
	E	S	S	S	N	S	N	S	N	S	N	N	N	N	N	N	
**29**	S	D	N	N	D	N	N	D	N	D	D	N	T	D	D	D	
	**C**	**G**	**A**	**A**	**U**	**A**	**C**	**G**	**C**	**G**	**U**	**C**	**C**	**U**	**U**	**U**	
	E	S	S	S	N	S	N	S	N	S	N	S	N	N	N	N	
**30**	S	D	N	N	D	N	N	D	N	D	N	N	T	N	D	D	
	**C**	**G**	**A**	**A**	**U**	**A**	**C**	**G**	**C**	**G**	**C**	**A**	**C**	**C**	**U**	**U**	
	E	S	S	S	N	S	N	S	N	S	N	S	N	N	N	N	
**31**	S	D	N	N	D	N	N	D	N	D	N	N	T	N	N	D	
	**C**	**G**	**A**	**A**	**U**	**A**	**C**	**G**	**C**	**G**	**C**	**A**	**C**	**C**	**C**	**U**	
	E	S	S	S	N	S	N	S	N	S	N	S	N	N	N	N	
**32**	S	D	N	N	D	N	N	D	N	D	N	N	T	D	D	D	
	**C**	**G**	**A**	**A**	**U**	**A**	**C**	**G**	**C**	**G**	**C**	**A**	**C**	**U**	**U**	**U**	
	E	S	S	S	N	S	N	S	S	S	N	S	N	N	N	N	
**33**	S	D	N	N	D	N	N	D	N	D	D	N	T	N	D	D	*nad7*
	**C**	**G**	**A**	**A**	**U**	**A**	**C**	**G**	**A**	**G**	**U**	**A**	**C**	**C**	**U**	**U**	
	E	S	S	S	N	S	N	S	S	S	N	S	N	N	N	N	
**34**	S	D	N	N	D	N	N	D	N	D	D	N	T	N	N	D	*nad7*
	**C**	**G**	**A**	**A**	**U**	**A**	**C**	**G**	**A**	**G**	**U**	**A**	**C**	**C**	**C**	**U**	
	E	S	S	S	N	S	N	S	S	S	N	S	N	N	N	N	
**35**	S	D	N	N	D	N	N	D	N	D	D	N	T	D	D	D	*ccmFc, nad7*
	**C**	**G**	**A**	**A**	**U**	**A**	**C**	**G**	**A**	**G**	**U**	**A**	**C**	**U**	**U**	**U**	
	E	S	S	S	N	S	N	S	S	S	N	S	N	N	N	N	
**36**	S	D	N	N	D	N	N	D	N	D	D	N	T	D	N	D	
	**C**	**G**	**A**	**A**	**U**	**A**	**C**	**G**	**A**	**G**	**U**	**A**	**C**	**U**	**C**	**U**	
	E	S	S	S	N	S	N	S	S	S	N	N	N	N	N	N	
**37**	S	D	N	N	D	N	N	D	N	D	D	N	T	N	D	D	
	**C**	**G**	**A**	**A**	**U**	**A**	**C**	**G**	**A**	**G**	**U**	**C**	**C**	**C**	**U**	**U**	
	E	S	S	S	N	S	N	S	S	S	N	N	N	N	N	N	
**38**	S	D	N	N	D	N	N	D	N	D	D	N	T	N	D	D	
	**C**	**G**	**A**	**A**	**U**	**A**	**C**	**G**	**A**	**G**	**U**	**C**	**C**	**C**	**U**	**U**	
	E	S	S	S	N	S	N	S	S	S	N	N	N	N	N	N	
**39**	S	D	N	N	D	N	N	D	N	D	D	N	T	N	N	D	
	**C**	**G**	**A**	**A**	**U**	**A**	**C**	**G**	**A**	**G**	**U**	**C**	**C**	**C**	**C**	**U**	
	E	S	S	S	N	S	N	S	S	S	N	N	N	N	N	N	
**40**	S	D	N	N	D	N	N	D	N	D	D	N	T	D	D	D	
	**C**	**G**	**A**	**A**	**U**	**A**	**C**	**G**	**A**	**G**	**U**	**C**	**C**	**U**	**U**	**U**	
	E	S	S	S	N	S	N	S	S	S	N	S	N	N	N	N	
**41**	S	D	N	N	D	N	N	D	N	D	N	N	T	N	N	D	*nad7*
	**C**	**G**	**A**	**A**	**U**	**A**	**C**	**G**	**A**	**G**	**C**	**A**	**C**	**C**	**C**	**U**	
	E	S	S	S	N	S	N	N	S	S	N	S	N	N	N	N	
**42**	S	N	N	N	N	N	N	D	N	D	D	N	T	N	D	D	*nad7, rps3*
	**C**	**A**	**A**	**A**	**C**	**A**	**C**	**U**	**A**	**G**	**U**	**A**	**C**	**C**	**U**	**U**	
	E	S	S	S	N	S	N	S	S	S	N	S	N	N	N	N	
**43**	S	N	N	N	D	N	N	D	N	D	N	N	T	D	N	D	*cox3, nad6,nad7*
	**C**	**A**	**A**	**A**	**U**	**A**	**C**	**G**	**A**	**G**	**C**	**A**	**C**	**U**	**C**	**U**	
	E	S	S	S	N	S	N	S	S	S	N	N	N	N	N	N	
**44**	S	N	N	N	D	N	N	D	N	D	N	N	T	D	D	D	*atp6, nad6, nad7*
	**C**	**A**	**A**	**A**	**U**	**A**	**C**	**G**	**A**	**G**	**C**	**C**	**C**	**U**	**U**	**U**	
	E	S	S	S	N	S	N	N	N	S	N	S	N	N	N	N	
**45**	S	N	N	N	D	N	N	D	N	D	N	N	T	N	N	D	
	**C**	**A**	**A**	**A**	**U**	**A**	**C**	**U**	**C**	**G**	**C**	**A**	**C**	**C**	**C**	**U**	

Motifs	P1	P2	P3	P4	P5	P6	P7	P8	P9	P10	P11	P12	P13	P14	P15	P16	Predicted target
	E	S	S	S	N	S	N	S	S	S	N	N	N	N	N	N	
**mRPF2**	S	N	N	N	D	N	N	D	N	D	N	N	T	D	N	D	*nad6*
	**C**	**A**	**A**	**A**	**U**	**A**	**C**	**G**	**A**	**G**	**C**	**C**	**C**	**T**	**C**	**U**	
	E	S	N	S	N	S	N	N	S	S	N	S	N	N	N	N	
**46**	S	N	N	N	D	N	N	D	N	D	D	N	T	N	D	D	
	**C**	**A**	**C**	**A**	**U**	**A**	**C**	**U**	**A**	**G**	**U**	**A**	**C**	**C**	**U**	**U**	
	E	S	N	S	N	S	N	N	S	S	N	S	N	N	N	N	
**47**	S	N	N	N	D	N	N	D	N	D	D	N	T	D	D	D	*nad5*
	**C**	**A**	**C**	**A**	**U**	**A**	**C**	**U**	**A**	**G**	**U**	**A**	**C**	**U**	**U**	**U**	
	E	S	N	S	N	S	N	N	S	S	N	S	N	N	N	N	
**48**	S	N	N	N	D	N	N	D	N	D	D	N	T	D	N	D	
	**C**	**A**	**C**	**A**	**U**	**A**	**C**	**U**	**A**	**G**	**U**	**A**	**C**	**U**	**C**	**U**	
	E	S	N	S	N	S	N	N	S	S	N	S	N	N	N	N	
**49**	S	N	N	N	D	N	N	D	N	D	N	N	T	N	D	D	
	**C**	**A**	**C**	**A**	**U**	**A**	**C**	**U**	**A**	**G**	**C**	**A**	**C**	**C**	**U**	**U**	
	E	S	N	S	N	S	N	S	N	S	N	S	N	N	N	N	
**50**	S	N	N	N	D	N	N	D	N	D	D	N	T	D	D	D	*nad5*
	**C**	**A**	**C**	**A**	**U**	**A**	**C**	**G**	**C**	**G**	**U**	**A**	**C**	**U**	**U**	**U**	
	E	S	N	S	N	S	N	S	S	S	N	S	N	N	N	N	
**51**	S	N	N	N	D	N	N	D	N	D	D	N	T	N	D	D	
	**C**	**A**	**C**	**A**	**U**	**A**	**C**	**G**	**A**	**G**	**U**	**A**	**C**	**C**	**U**	**U**	
	E	S	N	S	N	S	N	S	S	S	N	S	N	N	N	N	
**52**	S	N	N	N	D	N	N	D	N	D	D	N	T	N	N	D	
	**C**	**A**	**C**	**A**	**U**	**A**	**C**	**G**	**A**	**G**	**U**	**A**	**C**	**C**	**C**	**U**	
	E	S	N	S	N	S	N	S	N	S	N	S	N	N	N	N	
**53**	S	N	N	N	D	N	N	D	N	D	N	N	T	D	D	D	*cox2*
	**C**	**A**	**C**	**A**	**U**	**A**	**C**	**G**	**C**	**G**	**C**	**A**	**C**	**U**	**U**	**U**	
	E	S	N	S	N	S	N	S	S	S	N	N	N	N	N	N	
**54**	S	N	N	N	D	N	N	D	N	D	D	N	T	N	D	D	
	**C**	**A**	**C**	**A**	**U**	**A**	**C**	**G**	**A**	**G**	**U**	**C**	**C**	**C**	**U**	**U**	
	E	S	N	S	N	S	N	S	S	S	N	N	N	N	N	N	
**55**	S	N	N	N	D	N	N	D	N	D	D	N	T	D	D	D	
	**C**	**A**	**C**	**A**	**U**	**A**	**C**	**G**	**A**	**G**	**U**	**C**	**C**	**U**	**U**	**U**	
	E	S	N	S	N	S	N	S	S	S	N	S	N	N	N	N	
**56**	S	N	N	N	D	N	N	D	N	D	N	N	T	D	D	D	
	**C**	**A**	**C**	**A**	**U**	**A**	**C**	**G**	**A**	**G**	**C**	**A**	**C**	**U**	**U**	**U**	
	E	S	N	S	N	S	N	N	S	S	N	S	N	N	N	N	
**57**	S	N	N	N	D	N	N	D	N	D	D	N	T	N	D	D	
	**C**	**A**	**C**	**A**	**U**	**A**	**C**	**U**	**A**	**G**	**U**	**A**	**C**	**C**	**U**	**U**	
	E	S	S	S	N	S	N	S	N	S	N	N	N	N	N	N	
**58**	S	N	N	N	D	N	N	D	N	D	D	N	T	D	N	D	
	**C**	**A**	**A**	**A**	**U**	**A**	**C**	**G**	**C**	**G**	**U**	**C**	**C**	**U**	**C**	**U**	
	E	S	S	S	N	S	N	S	S	S	N	S	N	N	N	N	
**59**	S	N	N	N	D	N	N	D	N	D	D	N	T	N	D	D	*nad7, mttB*
	**C**	**A**	**A**	**A**	**U**	**A**	**C**	**G**	**A**	**G**	**U**	**A**	**C**	**C**	**U**	**U**	
	E	S	S	S	N	S	N	S	S	S	N	S	N	N	N	N	
**60**	S	N	N	N	D	N	N	D	N	D	D	N	T	D	D	D	*ox3, nad6, nad*
	**C**	**A**	**A**	**A**	**U**	**A**	**C**	**G**	**A**	**G**	**U**	**A**	**C**	**U**	**U**	**U**	
	E	S	S	S	N	S	N	S	S	S	N	S	N	N	N	N	
**61**	S	N	N	N	D	N	N	D	N	D	D	N	T	D	N	D	*ad6, nad7, mtt*
	**C**	**A**	**A**	**A**	**U**	**A**	**C**	**G**	**A**	**G**	**U**	**A**	**C**	**U**	**C**	**U**	
	E	S	S	S	N	S	N	S	S	S	N	N	N	N	N	N	
**62**	S	N	N	N	D	N	N	D	N	D	D	N	T	D	D	D	*atp1, nad6*
	**C**	**A**	**A**	**A**	**U**	**A**	**C**	**G**	**A**	**G**	**U**	**C**	**C**	**U**	**U**	**U**	
	E	S	S	S	N	S	N	S	S	S	N	N	N	N	N	N	
**63**	S	N	N	N	D	N	N	D	N	D	D	N	T	D	N	D	*nad6*
	**C**	**A**	**A**	**A**	**U**	**A**	**C**	**G**	**A**	**G**	**U**	**C**	**C**	**U**	**C**	**U**	
	E	S	S	S	N	S	N	S	S	S	N	N	N	N	N	N	
**64**	S	N	N	N	D	N	N	D	N	D	N	N	T	N	N	D	*nad6, nad7*
	**C**	**A**	**A**	**A**	**U**	**A**	**C**	**G**	**A**	**G**	**C**	**C**	**C**	**C**	**C**	**U**	
	E	S	S	S	N	S	N	N	S	S	N	N	N	N	N	N	
**65**	S	N	N	N	D	N	N	D	N	D	N	N	T	D	D	D	*tp1, nad6, nad*
	**C**	**A**	**A**	**A**	**U**	**A**	**C**	**U**	**A**	**G**	**C**	**C**	**C**	**U**	**U**	**U**	
	E	S	N	S	N	N	N	N	S	S	N	N	N	N	N	N	
**66**	S	D	N	N	N	N	N	D	N	D	N	N	T	N	N	D	
	**C**	**G**	**C**	**A**	**C**	**C**	**C**	**U**	**A**	**G**	**C**	**C**	**C**	**C**	**C**	**U**	
	E	S	S	S	N	N	N	S	S	S	N	S	N	N	N	N	
**67**	S	D	N	N	N	N	N	D	N	D	D	N	T	D	D	D	
	**C**	**G**	**A**	**A**	**C**	**C**	**C**	**G**	**A**	**G**	**U**	**A**	**C**	**U**	**U**	**U**	
	E	S	S	S	N	N	N	N	N	S	N	S	N	N	N	N	
**68**	S	D	N	N	D	N	N	D	N	D	D	N	T	D	N	D	
	**C**	**G**	**A**	**A**	**U**	**C**	**C**	**U**	**C**	**G**	**U**	**A**	**C**	**U**	**C**	**U**	
	E	S	S	S	N	N	N	S	S	S	N	S	N	N	N	N	
**69**	S	D	N	N	D	N	N	D	N	D	D	N	T	N	D	D	
	**C**	**G**	**A**	**A**	**U**	**C**	**C**	**G**	**A**	**G**	**U**	**A**	**C**	**C**	**U**	**U**	
	E	S	S	S	N	S	N	S	S	S	N	S	N	N	N	N	
**70**	S	D	N	N	D	N	N	D	N	D	N	N	T	D	D	D	*nad7*
	**C**	**G**	**A**	**A**	**U**	**A**	**C**	**G**	**A**	**G**	**C**	**A**	**C**	**U**	**U**	**U**	
	E	S	S	S	N	S	N	S	S	S	N	N	N	N	N	N	
**71**	S	N	N	N	N	N	N	D	N	D	D	N	T	D	N	D	*nad6*
	**C**	**A**	**A**	**A**	**C**	**A**	**C**	**G**	**A**	**G**	**U**	**C**	**C**	**U**	**C**	**U**	
	E	S	N	S	N	N	N	N	N	S	N	N	N	N	N	N	
**72**	S	D	N	N	N	N	N	D	N	D	D	N	T	D	N	D	
	**C**	G	C	A	C	C	C	**U**	**C**	**G**	U	C	C	U	C	U	
	E	S	N	S	N	N	N	S	S	S	N	S	N	N	N	N	
**73**	S	D	N	N	N	N	N	D	N	D	D	N	T	N	D	D	
	**C**	**G**	**C**	**A**	**C**	**C**	**C**	**G**	**A**	**G**	**U**	**A**	**C**	**C**	**U**	**U**	
	E	S	N	S	N	N	N	S	S	S	N	S	N	N	N	N	
**74**	S	D	N	N	N	N	N	D	N	D	D	N	T	D	D	D	
	**C**	**G**	**C**	**A**	**C**	**C**	**C**	**G**	**A**	**G**	**U**	**A**	**C**	**U**	**U**	**U**	
	E	S	N	S	N	N	N	S	S	S	N	S	N	N	N	N	
**75**	S	D	N	N	N	N	N	D	N	D	D	N	T	D	D	D	
	**C**	**G**	**C**	**A**	**C**	**C**	**C**	**G**	**A**	**G**	**U**	**A**	**C**	**U**	**U**	**U**	
	E	S	N	S	N	S	N	N	S	S	N	N	N	N	N	N	
**76**	S	D	N	N	N	N	N	D	N	D	D	N	T	N	D	D	
	**C**	**G**	**C**	**A**	**C**	**A**	**C**	**U**	**A**	**G**	**U**	**C**	**C**	**C**	**U**	**U**	
	E	S	N	S	N	S	N	N	S	S	N	S	N	N	N	N	
**77**	S	D	N	N	D	N	N	D	N	D	N	N	T	D	D	D	
	**C**	**G**	**C**	**A**	**U**	**A**	**C**	**U**	**A**	**G**	**C**	**A**	**C**	**U**	**U**	**U**	
	E	S	N	S	N	S	N	S	N	S	N	S	N	N	N	N	
**78**	S	D	N	N	D	N	N	D	N	D	D	N	T	N	D	D	
	**C**	**G**	**C**	**A**	**U**	**A**	**C**	**G**	**C**	**G**	**U**	**A**	**C**	**C**	**U**	**U**	
	E	S	S	S	N	N	N	N	S	S	N	N	N	N	N	N	
**79**	S	D	N	N	N	N	N	D	N	D	D	N	T	D	N	D	
	**C**	**G**	**A**	**A**	**C**	**C**	**C**	**U**	**A**	**G**	**U**	**C**	**C**	**U**	**C**	**U**	
	E	S	S	S	N	N	N	S	S	S	N	S	N	N	N	N	
**80**	S	D	N	N	N	N	N	D	N	D	D	N	T	D	N	D	
	**C**	**G**	**A**	**A**	**C**	**C**	**C**	**G**	**A**	**G**	**U**	**A**	**C**	**U**	**C**	**U**	
	E	S	S	S	N	S	N	N	S	S	N	S	N	N	N	N	
**81**	S	D	N	N	N	N	N	D	N	D	N	N	T	N	D	D	*rps3, nad7*
	**C**	**G**	**A**	**A**	**C**	**A**	**C**	**U**	**A**	**G**	**C**	**A**	**C**	**C**	**U**	**U**	
	E	S	S	S	N	S	N	S	N	S	N	S	N	N	N	N	
**82**	S	D	N	N	N	N	N	D	N	D	D	N	T	N	D	D	
	**C**	**G**	**A**	**A**	**C**	**A**	**C**	**G**	**C**	**G**	**U**	**A**	**C**	**C**	**U**	**U**	
	E	S	S	S	N	S	N	S	S	S	N	S	N	N	N	N	
**83**	S	D	N	N	N	N	N	D	N	D	D	N	T	N	N	D	*nad7*
	**C**	**G**	**A**	**A**	**C**	**A**	**C**	**G**	**A**	**G**	**U**	**A**	**C**	**C**	**C**	**U**	
	E	S	S	S	N	S	N	S	S	S	N	S	N	N	N	N	
**84**	S	D	N	N	N	N	N	D	N	D	D	N	T	D	N	D	
	**C**	**G**	**A**	**A**	**C**	**A**	**C**	**G**	**A**	**G**	**U**	**A**	**C**	**U**	**C**	**U**	
	E	S	S	S	N	N	N	S	N	S	N	N	N	N	N	N	
**85**	S	D	N	N	D	N	N	D	N	D	D	N	T	D	D	D	
	**C**	**G**	**A**	**A**	**U**	**C**	**C**	**G**	**C**	**G**	**U**	**C**	**C**	**U**	**U**	**U**	
	E	S	S	S	N	N	N	S	S	S	N	S	N	N	N	N	
**86**	S	D	N	N	D	N	N	D	N	D	D	N	T	D	N	D	
	**C**	**G**	**A**	**A**	**U**	**C**	**C**	**G**	**A**	**G**	**U**	**A**	**C**	**U**	**C**	**U**	
	E	S	N	S	N	N	N	N	S	S	N	S	N	N	N	N	
**87**	S	N	N	N	N	N	N	D	N	D	D	N	T	D	D	D	
	**C**	**A**	**C**	**A**	**C**	**C**	**C**	**U**	**A**	**G**	**U**	**A**	**C**	**U**	**U**	**U**	
	E	S	N	S	N	N	N	S	S	S	N	S	N	N	N	N	
**88**	S	N	N	N	N	N	N	D	N	D	D	N	T	D	N	D	
	**C**	**A**	**C**	**A**	**C**	**C**	**C**	**G**	**A**	**G**	**U**	**A**	**C**	**U**	**C**	**U**	
	E	S	N	S	N	S	N	S	S	S	N	N	N	N	N	N	
**89**	S	N	N	N	D	N	N	D	N	D	N	N	T	D	D	D	
	**C**	**A**	**C**	**A**	**U**	**A**	**C**	**G**	**A**	**G**	**C**	**C**	**C**	**U**	**U**	**U**	
	E	S	S	S	N	S	N	N	S	S	N	S	N	N	N	N	
**90**	S	N	N	N	D	N	N	D	N	D	D	N	T	D	D	D	*atp1, rps3*
	**C**	**A**	**A**	**A**	**U**	**A**	**C**	**U**	**A**	**G**	**U**	**A**	**C**	**U**	**U**	**U**	

Table S3. 177 transformants categorised by phenotype observed.

Variant number	Transformant number	Phenotype
**1**	5, 6	No visible phenotype
**2**	3,4	No visible phenotype
**3**	11, 12, 15, 18	No visible phenotype
**4**	1, 2, 236	No visible phenotype
**5**	8, 233, 235	No visible phenotype
**6**	9, 22	Dark green leaves and purple hypocotyls
**7**	24	No visible phenotype
**9**	226, 229, 230	No visible phenotype
**11**	225, 227, 234, 237	No visible phenotype
**12**	222, 223, 224	No visible phenotype
**13**	36, 41	Severe growth defect
**14**	25, 28, 30	No visible phenotype
**16**	38	Dark green leaves and purple hypocotyls
	37, 40, 55, 65, 68, 71, 72, 240, 241, 242	Slow growth
**17**	247, 249, 250	No visible phenotype
**18**	26, 27, 31, 33	No visible phenotype
**19**	251, 254, 255	No visible phenotype
**20**	29, 32, 244, 245, 246	Slow growth
**23**	252, 257	No visible phenotype
**24**	35	No visible phenotype
**25**	243	No visible phenotype
**26**	253, 256	No visible phenotype
**30**	43, 44	No visible phenotype
**34**	50, 52	Severe growth defect
	58, 66, 73	Small with curved leaves
**37**	49, 63, 69	Dark green leaves and purple hypocotyls
**39**	74, 91, 92, 286	No visible phenotype
**41**	287, 289	No visible phenotype
**42**	83	Dark green leaves and purple hypocotyls
**43**	285	No visible phenotype
**44**	291	No visible phenotype
**45**	42, 45, 47 48, 125, 263, 264, 266	Green leaves but slightly transparent
**46**	76, 87, 89, 90	No visible phenotype
**48**	283	No visible phenotype
**49**	78, 85, 86, 93	No visible phenotype
**50**	124	Small with curved leaves
**51**	77	No visible phenotype
**52**	84	No visible phenotype
**53**	303	No visible phenotype
**54**	305	No visible phenotype
**55**	119	No visible phenotype
**56**	302	No visible phenotype
**57**	304	No visible phenotype
**58**	114	No visible phenotype
**59**	101, 113	No visible phenotype
**60**	107, 108, 112	No visible phenotype
**61**	105, 110, 111	No visible phenotype
**62**	122, 126, 127	No visible phenotype
**63**	123	Severe growth defect
**64**	199, 200, 201, 202, 206, 308, 309, 310	Slow growth

Variant number	Transformant number	Phenotype
**65**	280	Slow growth
**67**	104, 204, 306	Dark green leaves and purple hypocotyls
**68**	103, 117	Small with curved leaves
**69**	232	Small with curved leaves
**71**	265, 293, 294	No visible phenotype
**72**	269	No visible phenotype
**73**	205, 295, 209	No visible phenotype
**76**	295	No visible phenotype
**78**	292	No visible phenotype
**80**	80, 267	No visible phenotype
**81**	238, 239	No visible phenotype
**83**	260, 261	No visible phenotype
**85**	51, 54, 56, 57, 59, 60, 67	Green leaves but slightly transparent.
**86**	100, 109, 115, 116, 120	Severe growth defect
**88**	79, 96, 99	Severe growth defect
	81, 82, 88, 288, 290, 296	Slow growth
**90**	121, 128, 217, 218, 219	Slow growth

Table S4. Primers used in this work.

Name	Sequence	Annealing temperature
RPF2 f2 pCAMBIA F	GGTAATGCTGCAACTGTTTCTCCC	58 °C
RPF2 pCAMBIA R	TGGCTGCAGGTCGACGGATCCTTAAGAAAGCATATC	58 °C
At5g09840.A	AAGACTGTGGTATGGTTCACCTG	60 °C
At5g09840.B	CTGAAACGAATCAGGCTATACCC	60 °C
At5g64710.A	ACAGGTTGTTCCTCTGGGAG	60 °C
At5g64710-komplR	CGTTTTGGTCAATTACCACGG	60 °C
RPF2 P2 R	CCATGGATTAGAGTGTTAAATGTGAC	64 °C
RPF2 P2 F	TGCAAAACTTGGGAATTCCTCAGA	64 °C
RPF2 P2mod F	GCAAAACTTGGGAATTCCTCAGG	64 °C
RPF2 P3 F	TTATGATTACAGAGAGAAACTGAGTA	58 °C
RPF2 P3 R	GTCGACAAAAACAGTTAATCAAAATCC	58 °C
RPF2 P3mod R	GTCGACAAAAACAGTTAATCAAAATCT	58 °C
RPF2 P5 R	TGAGGGAGCTGTAGGTAAC	64 °C
RPF2 P5 F	TTGCAAAAGGTTGTCAACCTG	64 °C
RPF2 P5mod F	GTTGCAAAAGGTTGTCAACCTA	64 °C
RPF2 P6 F	ATTGTCACGCTTTCTTCGCTGC	60 °C
RPF2 P6 R	TTACATAATCCATTTACTACCACCC	60 °C
RPF2 P6mod R	CTTACATAATCCATTTACTACCACCT	60 °C
RPF2 P8 R	TACTTGCAAAATCCCTTTATAAGC	58 °C
RPF2 P8 F	TGAGAGGAAAATCAACCCGG	58 °C
RPF2 P8mod F	ATTGAGAGGAAAATCAACCCGA	58 °C
RPF2 P9 F	TCATTGATGGTCTTTGCAAATACAAA	56 °C
RPF2 P9 R	ACAAACGCATCGATCAATGCTC	56 °C
RPF2 P9mod R	CACAAACGCATCGATCAATGCTT	56 °C
RPF2 P11 R	CCTTGCACATCCCTTCAATCATA	56 °C
RPF2 P11 F	GTCTCAAAGAGGATTGGTTGGAA	56 °C
RPF2 P11mod F	TCTCAAAGAGGATTGGTTGGAG	56 °C
RPF2 P12 F	ATTGTCACATACAGTTCATTGATCAA	60 °C
RPF2 P12 R	CTTGAAATAACCCTTGGATAAGAATTT	60 °C
RPF2 P12mod R	TTGAAATAACCCTTGGATAAGAATTC	60 °C
RPF2 P14 R	ATTAGTGACCAAGCCAATGGTTG	64 °C

Name	Sequence	Annealing temperature
RPF2 P14 F	GCCTTAAAGGAGTGAAGCCTG	64 °C
RPF2 P14mod F	AGCCTTAAAGGAGTGAAGCCTA	64 °C
RPF2 P15 F	ATGACATACAACACTTTGTTAGATG	64 °C
RPF2 P15 R	CTACAAAATCCTGATATCATTGTCT	64 °C
RPF2 P15mod R	CTACAAAATCCTGATATCATTGTCC	64 °C
RT1R	GGGGCTTTCACTTCGACACGT	58 °C
RT5R	TTTAAGGGCTTGTAGTAATTCAGGTTT	58 °C
cRT2F	GGTGGATTAACTAACGAAAGAAAAATGGAAC	58 °C
cRT3F	GAGGTGCAAGGCTGACAGAAGTAC	58 °C
cRT1R	GGCCATTCCTTTCACACCGTTGG	58 °C
cRT6R	ACACTATTTRGAATGGCTTTCTCATATTG	58 °C

Table S5. Antibodies used in this work.

Primary antibody	Dilution	Secondary antibody	Dilution	Origin
Nad9	1/50,000	anti-rabbit (Sigma A0545)	1/10,000	(Lamattina, 1993)
RISP	1/4000	anti-rabbit (Sigma A0545)	1/10,000	(Carrie et al., 2010)
Atp1	1/1000	anti-mouse (Sigma A4416)	1/10,000	Tom Elthon, University of Nebraska

Table S6. Untargeted proteomics data statistics.

AGI	s280_l2fc	adjusted_pvalue
**AT1G21400**	0.9338030354384730	0.21804093186244400
**AT2G26800**	−0.29738080364464400	0.9883638429358420
**AT2G33220**	−0.0310663545440096	0.991501251364609
**AT4G28220**	0.031447599882331100	0.993028358601147
**AT4G20760**	0.16443225404087100	0.9883638429358420
**AT4G20930**	0.5021731434698690	0.8327267791575600
**AT4G05590**	−0.10335862258465700	0.991501251364609
**AT5G14780**	−0.6481317663788740	0.017986908778417300
**AT5G12290**	−0.30603779476640000	0.9883638429358420
**AT5G43430**	−0.48133016542536900	0.3630280458982520
**AT5G26860**	0.6666254725304850	0.7978231723861180
**AT1G11860**	−0.07461220974557220	0.9883638429358420
**ATMG00160**	0.7699179452776850	0.927017151025293
**ATMG00070**	0.319734731024532	0.9883638429358420
**ATMG01190**	−2.4568018148834100	0.0007878992888031250
**ATMG00510**	−0.3683873833635770	0.6752979042618500
**ATMG00640**	−4.3336921047365000	0.007126829578467520
**AT5G15320**	0.22093582274131400	0.9883638429358420
**AT2G14170**	0.8751562430852330	0.16349424676117100
**AT3G15640**	−0.4206647341018490	0.9568240905387560

AGI	s280_l2fc	adjusted_pvalue
**AT4G21105**	0.32488954819305300	0.48125885609717400
**AT4G32210**	−1.2126439249484200	0.005663119366897890
**AT5G37510**	−0.1584195245493320	0.591376311508894
**AT3G59760**	−0.000764286325456295	0.9995992699999410
**AT1G53580**	−0.650087233773992	0.419643024235721
**AT1G55160**	−0.2804626598637020	0.6628265681601790
**AT1G17290**	0.40136546208195800	0.6691102220747460
**AT1G65540**	0.6006592449967150	0.5421067890148780
**AT1G19140**	−2.522508664599010	0.26367054053712500
**AT3G27570**	−0.9281147134592960	0.5748884032308050
**AT3G55410**	−0.06133501363040500	0.9883638429358420
**AT3G52200**	0.08340612352655440	0.991501251364609
**AT3G60810**	−1.031919975208080	0.04034764731501810
**AT3G07770**	1.3180007171452700	0.04034764731501810
**AT4G08390**	0.02826584634083640	0.991501251364609
**AT4G01100**	−1.5410767505104300	0.11875769430993200
**AT4G16800**	−0.5967474377899540	0.3313151906568340
**AT4G26860**	1.2223879021645200	0.6518369180034060
**AT5G41970**	−0.9481278295528480	0.5283465686382770
**AT5G55610**	0.145129377112597	0.9883638429358420
**AT3G16480**	1.0727759890013400	0.15283897489619300
**AT5G40770**	0.4618484590482030	0.5283465686382770
**AT4G02580**	0.208302640211462	0.8533944033685440
**AT2G43400**	0.118811492007645	0.991501251364609
**AT1G22840**	0.6592940729479040	0.14887879114463500
**AT4G36500**	−1.5586667301549600	0.3072136917017290
**AT2G42210**	0.15365032390512900	0.9883638429358420
**AT5G44730**	−1.3277563419353600	0.3567994520091640
**AT4G11600**	−0.8657301882555750	0.04034764731501810
**AT4G11010**	−0.39292659279884600	0.0971196810287904
**AT4G28390**	−0.14558719589214000	0.991501251364609
**AT4G28510**	0.49169484770295300	0.01730372307577340
**AT5G65720**	0.3781296653624980	0.5779381849857150
**AT4G22220**	0.18291926242229600	0.7602951265676660

AGI	s280_l2fc	adjusted_pvalue
**AT4G22310**	−0.047875338699256500	0.993028358601147
**AT2G19080**	−0.03025952363085610	0.993028358601147
**AT1G79230**	−0.25623833413009400	0.8444642106867830
**AT2G35010**	−0.12010908224599300	0.9883638429358420
**AT1G65290**	0.4258171603556250	0.31746248120533400
**AT2G26080**	0.32039528900330600	0.591376311508894
**AT3G10920**	−0.07683008112294820	0.927017151025293
**AT3G54110**	0.28030215137307600	0.41269786205678700
**AT2G35120**	0.14927951305140300	0.9883638429358420
**AT2G19680**	0.6755371116780960	0.8533944033685440
**AT5G63400**	−0.033596795009225700	0.991501251364609
**AT2G20420**	−0.2873004137575150	0.5020007954088740
**AT5G66760**	−0.059558314139658	0.991501251364609
**AT2G44350**	−2.230984688750180	0.5421067890148780
**AT2G35370**	0.016337665753567900	0.991501251364609
**AT3G23990**	0.282554863395287	0.04034764731501810
**AT3G08580**	−0.1974881688233550	0.5322551821141010
**AT5G23300**	0.6871538480487120	0.2942745100430790
**AT1G14980**	0.06518520477212950	0.8275511548435920
**AT1G50200**	−0.9705456239021540	0.26324709099990900
**AT5G13490**	−0.4040053908104300	0.6455782214935030
**AT4G08900**	−0.8301579379739700	0.6561906437612870
**AT2G30970**	0.06822328529742160	0.991501251364609
**AT1G59900**	−0.417501271589698	0.36229458343901100
**AT2G44620**	0.6120867327121190	0.31746248120533400
**AT5G08300**	0.004411512427008550	0.9995992699999410
**AT1G27390**	0.06338421327915740	0.993028358601147
**AT5G20080**	0.18664876376834900	0.8444642106867830
**AT5G08680**	−2.3401066544561600	0.002208306294219040
**AT3G30775**	0.4791159364255200	0.419643024235721
**AT2G17130**	1.763542534627630	0.591376311508894
**AT2G47510**	0.08991328920144070	0.927017151025293
**AT1G14610**	−1.2790084373348600	0.5315762656298350
**AT3G03060**	0.8258298930485350	0.4222090433702910

AGI	s280_l2fc	adjusted_pvalue
**AT1G15120**	−2.4044571742111500	0.4808100066522600
**AT4G00026**	−0.052000451084472200	0.991501251364609
**AT1G01170**	0.2829766180757860	0.9883638429358420
**AT4G27740**	−0.4907247821870710	0.9568240905387560
**AT5G50850**	0.005302873569702860	0.993028358601147
**AT1G72750**	0.11092336420001600	0.991501251364609
**AT4G00860**	0.12782725575807300	0.9883638429358420
**AT3G56070**	0.7566865583294470	0.1381368426899020
**AT5G07440**	−0.3217275552104110	0.9883638429358420
**AT3G22370**	3.0820292830779400	0.020785933386773500
**AT5G62575**	−0.09201315580177800	0.9883638429358420
**AT3G02090**	−0.1833952092980350	0.9883638429358420
**AT1G03090**	−0.3623531591902420	0.6691102220747460
**AT4G29130**	0.0757609236642131	0.991501251364609
**AT4G35830**	−0.7529418035539420	0.8444642106867830
**AT5G11770**	0.30272787076035400	0.9883638429358420
**AT1G79010**	−0.057885833360843500	0.991501251364609
**AT5G18170**	−0.2977087242285600	0.9883638429358420
**AT1G54220**	−0.055572500018363600	0.991501251364609
**AT2G36070**	0.796236796136534	0.31746248120533400
**AT4G11120**	3.2457087109397400	0.16349424676117100
**AT4G25280**	0.22952999564676700	0.9883638429358420
**AT4G26410**	0.11359259882439200	0.991501251364609
**AT4G16450**	0.03421729977333110	0.991501251364609
**AT2G29080**	2.5550403013048100	0.5283465686382770
**AT4G37910**	2.2752148153859200	0.002095865592896950
**AT1G72170**	−0.31899961951960300	0.9883638429358420
**AT5G48030**	3.43919114223241	0.21804093186244400
**AT5G51740**	−0.1660558218226490	0.9883638429358420
**AT4G26910**	−0.010723021479863100	0.9995992699999410
**AT1G24180**	−0.008401782226847550	0.9995992699999410
**AT2G33210**	0.7168459804326120	0.12531020801277000
**AT4G32605**	0.6413733724432300	0.419643024235721
**AT4G00570**	0.47154805173760900	0.9568240905387560

AGI	s280_l2fc	adjusted_pvalue
**AT5G61220**	0.6851132786610400	0.5407768269802510
**AT1G68680**	−1.506579925998330	0.5283465686382770
**AT5G23250**	0.48110836419551400	0.9883638429358420
**AT1G22520**	−0.23335288884826500	0.8444642106867830
**AT5G40650**	−0.905107128240928	0.591376311508894
**AT4G26780**	1.1481810473108800	0.05315817327314910
**AT3G15660**	0.29140852445559800	0.6628265681601790
**AT3G27380**	−0.371501584161298	0.8533983103396850
**AT3G45770**	−0.36869194661767700	0.8979217764257000
**AT1G23100**	1.7386268821635400	0.31746248120533400
**AT1G76200**	−0.5463594784875170	0.9883638429358420
**AT5G24165**	0.01810926055964820	0.993028358601147
**AT1G06130**	0.02783521340412010	0.993028358601147
**AT5G14105**	−0.9514371698684950	0.5748884032308050
**AT4G35260**	0.21429010771895000	0.9883638429358420
**AT5G27760**	−1.3322007242627400	0.16349424676117100
**AT3G09810**	1.211130422522520	0.06892937909869340
**AT3G06310**	0.8281513021270840	0.5962364607054960
**AT5G39410**	0.5798228522546760	0.9883638429358420
**AT5G14590**	−0.5396550146483310	0.9883638429358420
**AT3G13930**	−0.055984322516732000	0.9883638429358420
**AT5G27540**	0.16317230457023300	0.991501251364609
**AT5G16930**	0.7370866480040700	0.8444642106867830
**AT2G33255**	−1.521075642465680	0.1972644966551410
**AT4G21090**	−0.5413518197376660	0.6150237829637830
**AT5G14220**	0.18339781660292100	0.9883638429358420
**AT3G08950**	0.21141634130888900	0.9883638429358420
**AT5G62530**	0.13766092673134100	0.991501251364609
**AT4G00585**	−0.23429671901473100	0.9883638429358420
**AT3G07568**	−0.40601122429705800	0.9568240905387560
**AT5G10730**	−0.6336785253686120	0.9883638429358420
**AT4G27585**	0.9612245595994330	0.21804093186244400
**AT2G35605**	0.036362934068443200	0.991501251364609
**AT5G46800**	0.40784722336950200	0.26367054053712500

AGI	s280_l2fc	adjusted_pvalue
**AT3G16700**	0.46430419728947600	0.9883638429358420
**AT5G63620**	−0.4374489860370970	0.5306454979134860
**AT3G13860**	0.5272626724291900	0.49370871837522100
**AT1G08480**	0.6603665716969150	0.8275511548435920
**AT5G03290**	0.0989286280487439	0.9883638429358420
**AT4G28060**	0.320463056486437	0.9883638429358420
**AT4G34700**	−0.2078477970228700	0.8533944033685440
**AT5G20090**	−0.2502357769151490	0.9215461764760640
**AT5G15910**	−0.2334378659892790	0.991501251364609
**AT4G26970**	0.22009253266615500	0.9883638429358420
**AT4G20150**	0.4331462170089110	0.9883638429358420
**AT5G53140**	−1.4469637332288400	0.005663119366897890
**AT5G66510**	−0.28976650077515900	0.934553347785108
**AT5G06580**	0.09537600302037730	0.991501251364609
**AT2G45060**	−0.22522822622707500	0.9075198620215030
**AT4G33010**	0.5062034590930560	0.9568240905387560
**AT3G18410**	−1.7019422715429700	0.5874840463662210
**AT3G22200**	−0.37319952741293600	0.8444642106867830
**AT5G13430**	0.12750083606961200	0.9883638429358420
**AT3G62530**	1.3908820947976600	0.21804093186244400
**AT2G40765**	−0.142854176057084	0.9891381582060300
**AT2G33040**	−2.8520615462316300	0.04034764731501810
**AT5G13450**	−3.201305668426280	0.0005744664529373400
**AT5G47030**	−2.023115687323050	0.3072136917017290
**AT1G51650**	−3.205166757891960	0.01730372307577340
**AT4G39690**	−0.03333583889429810	0.991501251364609
**AT3G47833**	0.35775260287512000	0.5226333177230830
**AT5G19760**	−0.16565447346589600	0.9727914329789140
**AT1G47260**	−0.1837774766759100	0.9883638429358420
**AT1G50940**	−0.710756701326678	0.04034764731501810
**AT1G32580**	0.35219411782500100	0.9883638429358420
**AT1G74230**	0.5114822884893070	0.591376311508894
**AT1G53000**	−0.1381995743947670	0.9883638429358420
**AT5G05520**	0.18675136638966300	0.991501251364609

AGI	s280_l2fc	adjusted_pvalue
**AT5G47570**	1.6551473663508200	0.6863814907707030
**AT5G51040**	1.2169349226414400	0.5283465686382770
**AT5G47890**	0.04026738040285330	0.991501251364609
**AT5G53650**	−0.4480410223198030	0.49370871837522100
**AT5G67590**	−0.06205524505699190	0.991501251364609
**AT5G67500**	−0.20761675793369600	0.9891381582060300
**AT5G53350**	3.4894487819268200	0.005876134347731720
**AT5G50370**	0.22617220995589000	0.9568240905387560
**AT5G57490**	0.06639411904115070	0.991501251364609
**AT3G10860**	−0.42690721239729100	0.6904854329885030
**AT5G65750**	−0.3362882712733270	0.8275511548435920
**AT5G55200**	2.781522857931240	0.16349424676117100
**AT5G55070**	−0.09183458082446950	0.991501251364609
**AT5G52840**	0.03357801596434370	0.991501251364609
**AT5G14040**	−0.002640302362123610	0.9995992699999410
**AT5G23140**	0.22676806259281700	0.9883638429358420
**AT5G43970**	−0.0767865420460329	0.9883638429358420
**AT5G46180**	0.47382729590508400	0.21804093186244400
**AT5G08530**	0.13242344403577500	0.991501251364609
**AT3G52300**	−3.038916071575180	$2.65932960014406 \times 10^{-5}$
**AT1G71310**	−0.5939697221552090	0.9684684946972320
**AT1G19580**	0.009739353681477010	0.9995992699999410
**AT1G67350**	2.0678070539921100	0.419643024235721
**AT1G26460**	1.108404431280960	0.3313151906568340
**AT3G03600**	−0.6498734922225140	0.591376311508894
**AT4G34030**	−2.7531684551621900	0.419643024235721
**AT5G09590**	0.47497302971429500	0.01755128092619030
**AT5G10860**	0.39165414618116200	0.9582978075448450
**AT3G20000**	0.5122986569098850	0.5121268941137440
**AT3G12260**	−0.11556895620393800	0.9883638429358420
**AT3G20970**	0.7720253082187830	0.5283465686382770
**AT3G27280**	2.413110221630090	0.024266879166692200
**AT3G27240**	0.20187948637740900	0.9883638429358420
**AT3G27890**	−0.9692917649550590	0.21804093186244400

AGI	s280_l2fc	adjusted_pvalue
**AT3G15090**	0.06106319512179120	0.9883638429358420
**AT3G15000**	1.076211562674090	0.05088360757421670
**AT1G32470**	0.1642272654071400	0.987798375992696
**AT3G22330**	0.5156394748206780	0.8444642106867830
**AT5G58270**	−0.052079820841084600	0.991501251364609
**AT5G54100**	2.8393114659133000	0.3072136917017290
**AT3G52730**	0.12311167003749500	0.9883638429358420
**AT3G56430**	0.0004932816288751120	0.9995992699999410
**AT2G47690**	0.7360651621572270	0.9568240905387560
**AT5G02050**	1.2518350909878400	0.023419736961663000
**AT3G62400**	0.20237139636595800	0.6150237829637830
**AT4G29480**	−0.03985347213748190	0.991501251364609
**AT4G05400**	1.2196688707463300	0.6455782214935030
**AT3G59820**	1.512366221347460	0.18388979708896700
**AT3G17240**	0.2356165686936250	0.764838075661307
**AT1G48030**	−0.08926756832157310	0.6455782214935030
**AT3G06050**	0.2538179301937290	0.9568240905387560
**AT3G06850**	0.4045729656966430	0.6150237829637830
**AT1G49140**	−0.20038238421814300	0.8874074447865420
**AT3G03100**	−0.12905707043365800	0.991501251364609
**AT1G14450**	−0.40506114405308500	0.5315762656298350
**AT3G01130**	−0.3661159530969590	0.9883638429358420
**AT3G61440**	0.25259300279786300	0.6987638389338800
**AT1G22450**	−0.1840483702333680	0.9883638429358420
**AT1G79440**	−0.0527309891467113	0.991501251364609
**AT4G01660**	0.7382497150718240	0.927017151025293
**AT5G40610**	0.0662936721967021	0.991501251364609
**AT5G08060**	−0.06195725810380670	0.991501251364609
**AT5G08040**	0.05502381181181600	0.991501251364609
**AT1G28510**	−0.8459535099421310	0.591376311508894
**AT1G06530**	−0.4741655149781230	0.11639743742969300
**AT2G05710**	0.1662419106668810	0.9883638429358420
**AT2G20530**	0.4870346715721260	0.591376311508894
**AT2G31490**	−0.7438170213400950	0.49370871837522100

AGI	s280_l2fc	adjusted_pvalue
**AT2G13560**	2.1096068193456200	0.6767826802850450
**AT2G16460**	0.5908442146721250	0.6863814907707030
**AT2G21870**	−3.4840040887662000	0.00042117448726334100
**AT2G20360**	0.058699082809786700	0.991501251364609
**AT2G02050**	−0.15028431850995000	0.8444642106867830
**AT2G28430**	−0.6434152516584090	0.9883638429358420
**AT3G48680**	0.4337014549516380	0.9568240905387560
**AT5G15090**	0.48917869014954200	0.6863814907707030
**AT3G46430**	−4.184771051403230	0.0015532596475782800
**AT3G01280**	0.1718617027942930	0.9883638429358420
**AT3G11070**	1.5012875261082400	0.6150237829637830
**AT3G07480**	0.2841909647331150	0.987798375992696
**AT3G10370**	−0.27270170296812600	0.927017151025293
**AT1G80230**	−0.09878587768415650	0.991501251364609
**AT4G12340**	0.20566678499286600	0.9883638429358420
**AT4G26210**	−0.10936431329786700	0.9883638429358420
**AT3G47930**	0.012564913520364500	0.993028358601147
**AT3G48000**	0.022707127751830300	0.9883638429358420
**AT4G32470**	0.016117892549638400	0.991501251364609
**AT4G13850**	−0.4139277914282880	0.49370871837522100
**AT3G45300**	−0.24207055948198600	0.5283465686382770
**AT1G47420**	−0.13166922276787600	0.9881796667235620
**AT1G04640**	−0.7614057725642930	0.419643024235721
**AT4G09750**	−0.05020286410489100	0.993028358601147
**AT4G37930**	0.7920983585122040	0.0012629909820399600
**AT4G30000**	−1.1701210526741000	0.21570307375042400
**AT4G30010**	−3.5475746244184100	0.0002294904219890030
**AT4G37830**	0.19242256123489100	0.991501251364609
**AT4G10040**	0.3501567360293450	0.9883638429358420
**AT4G13360**	−0.8082931416937960	0.5421067890148780
**AT3G46560**	1.3499869048032700	0.671420390193162
**AT5G50810**	1.4020982330617500	0.8393093113240710
**AT5G25940**	−0.5516907820927550	0.8444642106867830
**AT1G61570**	0.7995311453842700	0.5283465686382770

AGI	s280_l2fc	adjusted_pvalue
**AT1G03860**	0.5475399057521680	0.5283465686382770
**AT1G53240**	−0.018147846232802000	0.991501251364609
**AT4G08870**	−0.5998066091505920	0.8170627155951060
**AT2G18330**	0.9696323644543040	0.24201686890069500
**AT2G46540**	−0.3697011188705390	0.49370871837522100
**AT4G03240**	0.2224478895598000	0.991501251364609
**AT4G02930**	0.24652602874659700	0.4357471946801780
**AT1G51980**	0.054724688971039800	0.991501251364609
**AT2G27730**	−0.46351477390039600	0.5283465686382770
**AT2G38670**	−0.270913827813111	0.9883638429358420
**AT2G29530**	0.20767327607404700	0.9891381582060300
